# Role-Playing Between Environmental Pollutants and Human Gut Microbiota: A Complex Bidirectional Interaction

**DOI:** 10.3389/fmed.2022.810397

**Published:** 2022-02-16

**Authors:** Federica Giambò, Chiara Costa, Michele Teodoro, Concettina Fenga

**Affiliations:** ^1^Occupational Medicine Section, Department of Biomedical and Dental Sciences and Morphofunctional Imaging, University of Messina, Messina, Italy; ^2^Clinical and Experimental Medicine Department, University of Messina, Messina, Italy

**Keywords:** microbiota, environmental pollutants, occupational medicine, chronic diseases, occupational toxicology

## Abstract

There is a growing interest in the characterization of the involvement of toxicant and pollutant exposures in the development and the progression of several diseases such as obesity, diabetes, cancer, as well as in the disruption of the immune and reproductive homeostasis. The gut microbiota is considered a pivotal player against the toxic properties of chemicals with the establishment of a dynamic bidirectional relationship, underlining the toxicological significance of this mutual interplay. In fact, several environmental chemicals have been demonstrated to affect the composition, the biodiversity of the intestinal microbiota together with the underlining modulated metabolic pathways, which may play an important role in tailoring the microbiotype of an individual. In this review, we aimed to discuss the latest updates concerning the environmental chemicals–microbiota dual interaction, toward the identification of a distinctiveness of the gut microbial community, which, in turn, may allow to adopt personalized preventive strategies to improve risk assessment for more susceptible workers.

## Introduction

During the entire lifespan, since the conception and whole fetal development, we are constantly exposed to the so-called exposome, defined as the totality of the environmental exposures, which are dynamic in their quality and quantity over time (1). Such environmental factors may include air pollutants, radiations, chemicals present in soil, food, and water, but also individual factors associated with the personal lifestyle, such as tobacco smoking, food consumption, specific use of drugs, and xenobiotics, altogether representing the external exposome (2).

Another constant source of exposure is considered our internal exposome, mainly accounting for the effects mediated by the microbiota, a heterogeneous consortium of microorganisms that populates all the exposed surfaces of the body, and having with the host a relationship of mutual advantage (3). For many chemicals, the health impact associated with exposure (and the corresponding exposure pathways) remains yet poorly understood (4). To find novel biomarkers of exposure, as well as to characterize the real associations existing between exposures and the development of a certain disease, both represent goals of pivotal importance, especially for the most exposed occupational categories (including agriculture, construction plant manufacturing, and mining) (5). Given its role as a key link between the external exposome and the human health, the internal exposome, and in particular our microbiota represents a promising source of novel functional correlations and biomarkers of exposure (6).

Among the different microbiota, the most characterized is the gastro-intestinal one, which plays a main role as a dynamical interface between the host and the external exposome (7). Intestinal dysbiosis has been associated with marked structural changes in the mucosa, including permeability and inflammation. The dysbiosis may, in turn, favor either the insurgence or the worsening of several chronic, non-communicable diseases, including cardiovascular diseases, diabetes, neurological diseases, and cancer (8, 9). Furthermore, dysbiosis has been associated with multiple extraintestinal diseases such as neurological or behavioral outcomes (10–14), respiratory dysfunctions (15, 16), metabolic/endocrine impairment (17–19), and inflammatory/autoimmune diseases (20–26).

Additionally, it is now commonly recognized that stress actively modulates both structure and activity of the Gut Microbiota (GM) community and it may be a critical factor in causing dysbiosis (27–30). There is a growing evidence that a healthy and resilient GM can contribute to optimize the health and performance of the general host. However, developing responses for this goal requires elucidating the impact of stressors on the GM. Environmental pollutants such as microplastics or synthetic compounds interact with GM through several toxicokinetic and toxicodynamic pathways and, in turn, GM may alter the bioavailability and toxicity of xenobiotic metabolites. This bidirectional interaction can modulate the physiological homeostasis, finally leading to health disorders (31–33).

This review explores all the up-to-date studies regarding the role played by the GM in xenobiotics metabolism and protection mechanisms, especially for what concerns the main groups of environmental chemicals (i.e., pesticides, metals, and microplastics) to tailor personalized preventive strategies, as well as to improve the risk assessment for more susceptible workers.

## Features of Gut Microbiota

GM includes bacteria, archaea, viruses, yeasts, and other fungi whose population density progressively increases from 10^3^ to 10^4^ cells/ml within the gastric acidic environment to about 10^11^ cells/ml within the colon (34). The dominant phyla are *Firmicutes, Bacteroidetes, Actinobacteria, Proteobacteria*, and *Fusobacteria*. Overall, a healthy human adult hosts over 100 different bacterial species in the gastrointestinal tract, with a marked interindividual variation in genus and species compositions (35).

The so-called gut microbiome includes the whole genome of the GM, encoding for over 100-fold more genes than the human genome (36). The recent advent of metagenomics, which combines the next-generation sequencing (NGS) technology with the computational analysis of the 16S ribosomal RNA (rRNA) amplicons, helps in the characterization of both diversity and abundance of the gut microbiome (37). Metagenomics, together with metatranscriptomics, metaproteomics, and metabolomics, is currently allowing us to understand the impact of each individual bacterial species on the health of the host (38).

Despite the existence of several definitions of healthy GM, a number of endogenous and exogenous factors may cause the microbiota to shift from eubiotic to dysbiotic; in general, a more diverse GM, both in terms of diversity and abundance of taxa, is considered a healthier GM (39). Additionally, a healthy GM can resist or overcome perturbations by returning to a state of balance or eubiosis.

Currently, species belonging to the *Eubacterium, Roseburia*, and *Faecalibacterium* genera are included among the beneficial taxa, given their ability to secrete butyrate, a short-chain fatty acid (SCFA) with several health effects, such as to improve the integrity of the intestinal barrier, as well as to reduce the gut oxidative stress or inflammatory status (40).

Potentially harmful bacteria are considered those belonging to the *Enterobacteriaceae* family that includes the intestinal commensals *Escherichia, Shigella, Proteus*, and *Klebsiella* with pronounced pro-inflammatory effects (41). Several bacterial species are able to actively secrete toxins, including CagA from *Helicobacter pylori*, colibactin and cytolethal distending toxin (CDT) from *Escherichia coli*, inositol phosphate phosphatase D (IpgD), and cysteine protease-like virulence gene A (VirA) from *Shigella flexneri*, that in turn damage the intestinal epithelial integrity (9). These toxins may induce direct damage to the epithelial cellular DNA, trigger proproliferative pathways (including Akt serine/threonine kinase family and Wnt/β-Catenin signaling), or stimulate the local secretion of reactive oxygen species (ROS). Overall, these toxins may promote a proinflammatory milieu and even trigger the local cellular neoplastic transformation (9).

The proinflammatory environment may also have a counterintuitive health effect on the human host. For example, the lipopolysaccharide (LPS, also known as endotoxin), the main component of the outer membrane in gram-negative bacteria, may activate the pattern recognition receptors (PRRs) of host, including the toll-like receptor 4 (TLR4), thereby activating the immune T cell-mediated response, which activates a solid proinflammatory reaction. Although the inflammation may often promote colitis or mucositis, a proinflammatory status may also be protective in certain conditions, such as during tumor development (42).

Specific intestinal taxa are able to secrete essential micronutrients, such as vitamins (i.e., vitamin K and vitamin B) or the linoleic acid, which is an antidiabetic compound. Also, specific bacteria can catabolize secondary bile acids and phenolic compounds (43). Finally, certain gut-resident taxa, upon fermentation of dietary fibers in the large intestine, may produce hormone-like metabolites, such as the SCFAs.

Additionally, the gut microbial functions are tightly interconnected and directly affect the host immune response, both locally and systemically (44). This topic is deeply analyzed elsewhere, and, although out from the scope of this review, it is important here to underline that the microbial dysbiosis deriving by both environmental exposure and genetic susceptibility may be associated with aberrant mucosal immune responses, such as the upregulation of the Th17, Th1, and Th2 immune phenotypes, the downregulation of the T regulatory cells, and dysregulated humoral immunity. Overall, this immune shift may result in chronic intestinal inflammation and a generally altered immune response to pathogens and insults (45).

**Figure 1:**
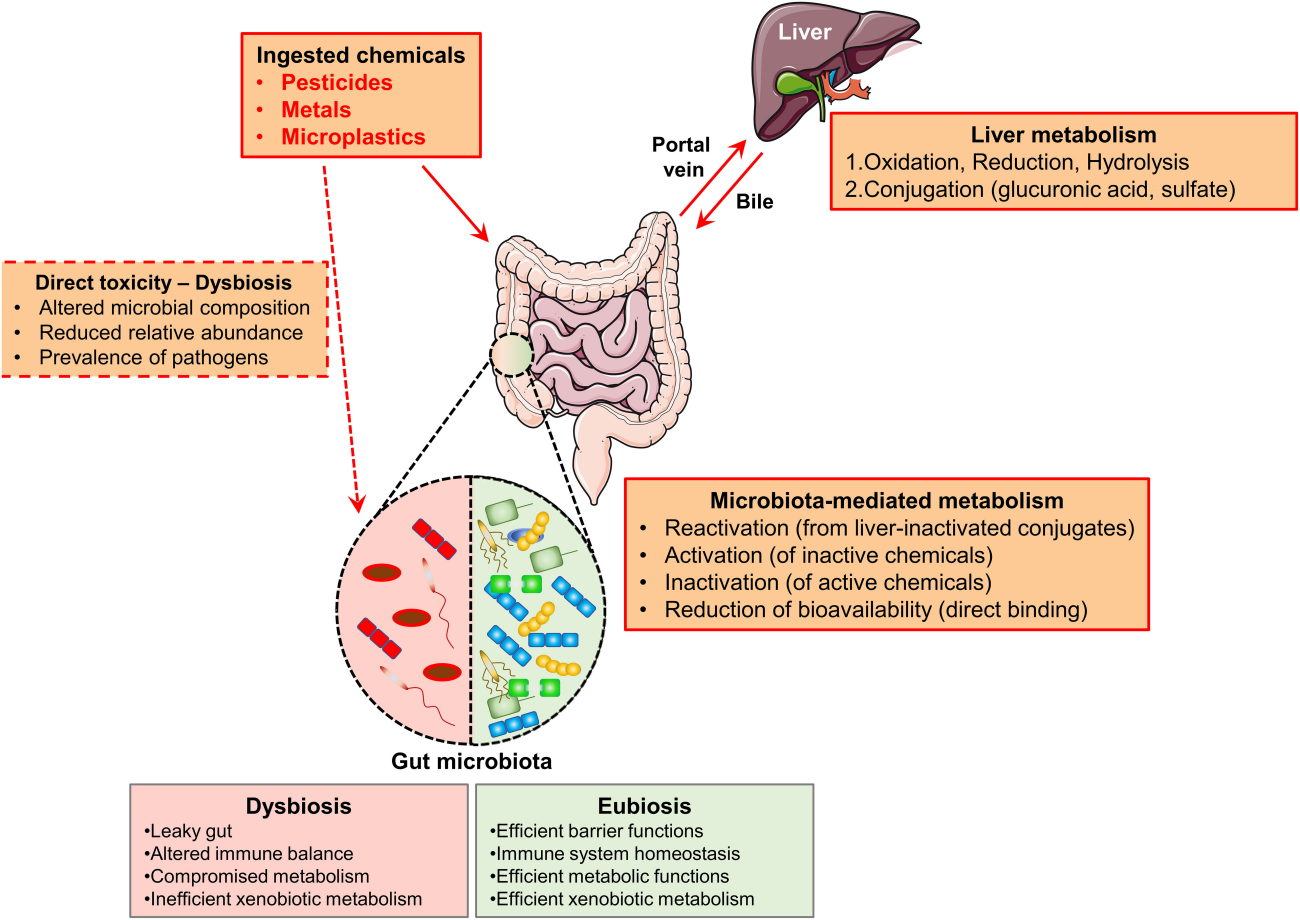
Schematic representation of the biotransformation routes of ingested chemicals. Ingested chemicals (pesticides, metals, microplastics), arrive in the intestine through the oral route. Well-adsorbed compounds are transported to the liver through the portal vein. In the liver, such compounds may be metabolized (through the action of liver enzymes, the compounds are oxidized, reduced, or hydrolysed and finally they are conjugated) and hence released in the intestine within the bile. The gut microbes can: reactivate conjugated chemicals, directly metabolize non-adsorbed chemicals (activation or inactivation), or direct bind such compounds (reducing their bioavailability). Importantly, several chemicals might directly induce microbial dysbiosis (red dotted box). The features of a dysbiotic or eubiotic intestinal microbiota are summarized, respectively, in the red and green boxes.

Given the role as a barrier, metabolic, and immune interface, the GM is extremely important as the joining link between the external exposome and the human host (46). In details, the GM plays a pivotal role in xenobiotics metabolism, including toxicants and chemical pollutants (47) (Figure 1).

Once ingested, the toxicants, which are efficiently adsorbed in the intestine, through the bloodstream, arrive at the liver where they are oxidized, forming conjugates with glucuronic acid, sulfate, or glutathione that can be excreted in the bile and enter the intestine again, where the GM may interfere with their excretion (47). The GM can additionally directly metabolize the chemicals which are poorly adsorbed and hence transported to the large intestine through the action of several bacterial enzymes (such as beta-glucosidases, beta-glucuronidases, sulfatases, azoreductases, nitroreductases, and transferases) (48). Overall, the GM-mediated metabolism can lead to: (1) inactivation, (2) activation, or (3) reactivation (upon liver inactivation) of the specific compound (49). In the case of toxicants such as chemical pollutants, the GM-mediated inactivation can neutralize the hazard of the exposure. On the contrary, the activator outcome may be detrimental and increase the risk of developing associated pathologies, including cancer (50). In parallel, the GM composition may be actively shaped by the chemicals, as it was demonstrated in a number of preclinical studies (51). Many examples will be further discussed in the following section.

Consequently, the maintenance of a healthy GM may be protective against the toxicity of such chemicals and the occurrence of associated chronic diseases (52). This could be very relevant in specific occupational settings with a high rate of specific exposures (53). Overall, the modulation of the intestinal gut microbes, through active strategies such as the assumption of specific nutrients or also specific beneficial probiotics, with the aim to repristinate the eubiosis, may be protective against the development of the linked diseases and it can be suggested as a preventive intervention method.

## Bidirectional Interaction Between Environmental Chemicals and Gut Microbiota

The concern is about the adverse health effects deriving from occupational exposure to toxic substances and environmental pollutants. Several xenobiotic chemicals have been described to interact with the biological activity of GM affecting the microbial composition and global homeostasis, with dangerous alterations to the host (48, 49). Noteworthy are occupational exposures to pesticides, used for the control of pests, which could affect human GM (54). Heavy metals (HMs) including cadmium, lead, arsenic, and other metals can contaminate soils and reach the human GM through the food chain (55). Exposures to environmental toxicants have been studied primarily for long-term systemic health effects on respiratory disease and cognition, among others, but there is growing evidence that these components also affect the GM. The molecular mechanisms leading to these interactions are not well-known. GM composition depends on several factors and significant changes in this composition, even if minor, are very expected to happen in studies involving animals and chemicals, but it is not clear if these alterations lead to biologically relevant outcomes in the host (56). Establishing such causal relationships should be a priority to elucidate this topic. Now we discuss recent literature findings of interaction between main environmental chemicals (pesticides, metals, and microplastics) and GM. The main findings are shown in Table 1.

**Table 1:** Main results of the studies included in this review.

**References**	**Experimental model**	**Pesticides**	**Microbiota changes**
Mao et al. (57)	Rats	Gly	*↑ Prevotella* *↑ Muscispirillum* *↓ Lactobacillus* *↑ Aggregatibacter*
Ruuskanen et al. (58)	Japanese quails	Gly	*↓ Firmicutes* *↑ Actinobacteria*
Krause et al. (59)	*In vitro*	Gly	*Not evident effects*
Ding et al. (60)	Zebrafish	Gly	*↑ Fusobacteria* *↓ Proteobacteria*
Tang et al. (61)	Rats	Gly	*↓ Firmicutes*
Nielsen et al. (62)	Rats	Gly	Not evident effects
Liang et al. (63)	Mice	Cpf	*↑ Proteobacteria* *↓ Bacteroidetes*
Joly Condette et al. (64)	Rats	Cpf	*↓ Lactobacillus* *↓ Bifidobacterium*
Reygner et al. (65)	SHIME	Cpf	*↑ Enterococcus* *↑ Bacteroides*
Alberoni et al. (66)	Honeybee	Imidacloprid	*↓ Firmicutes*
		**Metals**	
Dong et al. (67)	Human	As	*↑ Proteobacteria*
Wang et al. (68)	Earthworm	As	*↑ Proteobacteria*
Eggers et al. (69)	Human	Pb	*↑ Proteobacteria*
Yu et al. (70)	*In vitro* and mice	Pb	*↓ Coprococcus* *↓ Oscillospira* *↑ Lactobacillus*
Podany et al. (71)	Mice	Zn	↑*Pseudomonadales* ↑ *Campylobacter*
		**Microplastics**	
Xie et al. (72)	Zebrafish	Mps	*↑ Proteobacteria*
Zhu et al. (73)	Soil animal	Mps	*↓ Bacteroides* *↑ Firmicutes*
Li et al. (74)	Mice	Polyethylene mps	*↑ Staphylococcus* *↓ Parabacteroides*
Wang et al. (75)	Bees	Polystyrene mps	*↓α-diversity*
Cheng et al. (76)	Earthworm	Polypropylene mps	*↑ Aeromonadaceae* *↑ Pseudomonadaceae* *↓ Nitrososphaeraceae* *↓ Proteobacteria*

### Pesticides

Many studies have focused on the mechanisms underlying the relationship between pesticides and GM (47, 77). The GM can metabolize pesticides after absorption and reciprocally, active metabolites can affect GM homeostasis with adverse effects for the host. Glyphosate (Gly) is the most widely used herbicide worldwide and its use has been related to several adverse outcomes in humans (48, 49). Gly-induced GM alteration has been hypothesized to be related to neurological impairment such as autism spectrum disorders (78, 79). Several studies showed that Gly can alter the abundances of gut microbial species both *in vivo* (57, 58, 80–82), *in vitro* (59), and also through bioinformatics tools (83). It has been estimated that more than half of species living in the central human GM are sensitive to Gly (84). In addition to altering the microbial composition, a possible mechanism of action could be the modification of microRNAs (miRNAs) expression and immunomodulation as suggested by several studies (85–87). The mRNA expression levels of several inflammatory mediators [Nuclear factor kappa B (NF-κB), Tumour necrosis factor α (TNF-α), Caspase-3, MAPK3, IL-1β, and IL-6] resulted in increase after exposure to Gly, highlighting a notable decrease in the abundance of Firmicutes and enhancement of pathogenic bacteria (60, 61). Nielsen et al. showed that the presence of aromatic amino acids could relieve the antimicrobial effect of Gly (62). Another commonly used pesticide is Chlorpyrifos (Cpf), an organophosphate insecticide effective against fruit and vegetable pests. *In vivo*, it was observed that a chronic exposure to Cpf could cause an increment in Proteobacteria phylum and a decrease in Bacteroidetes phylum (63). The effect of chlorpyrifos has been explored using an *in vitro* simulator mimicking the human intestinal environment. It was observed a reduction of the *Lactobacillus* and the *Bifidobacterium* counts and alteration of the epithelial barrier integrity (64, 88) although other authors described an increment in the cultured *Enterococcus* spp. and *Bacteroides* spp. counts (65). Honeybees seem to be severely affected by neonicotinoid insecticides such as Imidacloprid and many studies suggested that a chronic exposure can alter normal GM composition with a decrement in the global bacterial count, mainly due to Firmicutes reduction (66, 89). Both Gly and Cpf can modulate mucosal-associated invariant T-cells activity in humans, leading to a pro-inflammatory immune response (90).

### Metals

Heavy metals include naturally occurring chemicals with high atomic weight and density. Typically, these chemicals can be conveyed with particulate matter especially in urban areas and then reach the water and the soil (91). Several studies on humans or *in vitro* models suggest that HMs exposure can alter the composition and integrity of the GM (92–95). The changes of diversity and composition profile of GM composition resulted altered after a chronic exposure to several metals, such as arsenic (As), cadmium (Cd), cuprum (Cu), lead (Pb), and zinc (Zn). The authors observed an increase in the counts of some families (*Porphyromonadaceae, Erysipelotrichaceae, Lachnospiraceae*, and *Acidaminococcaceae*) vs. a reduction of the Prevotellaceae family. Moreover, it was found a gender difference because microbiota alterations of men were associated with work activity (mining and smelting) in polluted areas (96). As is common chemicals in nature, defined as a human carcinogen since 2012 (97). It can be found in water and soil, as both the organic and inorganic structures. It has been demonstrated to shape the GM depleting gut commensals and enriching pathogenic bacteria (98). In children exposed to As the GM alteration resulted in an abundance of *Proteobacteria*, highlighting changes in genes involved in multidrug resistance (67). As exposure not only affects GM composition but can also alter immune response increasing inflammatory cytokines such as IL-17, TNF-α, and interferon-γ (IFN-γ) (99). As can induce shifts in the earthworm GM, increasing the counts of *Proteobacteria*; these effects are amplified in a synergistic manner in a mixture exposure of As and microplastics (MPs) (68). Other authors described this combined effect of MPs and HMs including Cd, Pb, and Zn underlining GM perturbation and gonadal development in aquatic organisms (100). Pb exposure has been associated with alterations in the composition of the adult GM in humans. Increased urinary Pb level was related to GM richness and α and β-diversity, remarking an increment of *Proteobacteria* (69). Cd determines a reduction in GM richness and SCFAs production in mice; additionally, it can change the expression of genes involved in several metabolic pathways (101). In amphibians Cd has been demonstrated to reduce GM biodiversity and arrangement, disclosing significant gut histological alteration at Cd exposure (100 and 200 μg/l) (102). Mercury (Hg) toxicity on GM is confirmed by several *in-vivo* studies. The main described effects are intestinal injury, dysbiosis, enhanced expression of apoptosis genes, and neurotoxic effects (103, 104). Similarly, Pb exposure may act as a disruptor in GM homeostasis, inducing structural intestinal injuries and perturbing GM diversity both *in vivo* and *in vitro* (70). A disproportionate diet introit of Zn in mice, has been shown to cause oxidative stress in the intestinal trait, accompanied by significant shifts in GM, such as enrichment in pathogenic taxa (71).

### Microplastics

Since plastics appeared, the MPs represent a novel occupational and environmental hazard (105). Although there is no scientifically agreed definition of MPs, they are usually defined as plastic particles <5 mm in diameter. Its toxicity has been broadly debated (106, 107), even though knowledge about MPs effects on gut microbiota still lacks. The accumulation of different forms of microplastics can cause several effects in the intestinal tract, damaging mucosa, and increasing permeability. Some authors have hypothesized that MPs could carrier and release phthalate esters into intestinal traits with consequential toxic effects (108). Also, MPs also can induce GM dysbiosis and specific bacteria alterations (109, 110). In the aquatic organism, MPs can affect the GM causing several harmful effects. Authors showed that low chronic MP exposure in mussel GM can lead to an increased abundance of human pathogens (111). In the zebrafish gut, it was observed a significant alteration in the microbial community after exposure to MPs. These alterations were likely mediated by inflammation and oxidative stress (112–114). At the phylum level, the increased count of *Proteobacteria* was accompanied by a significant reduction in the count of *Fusobacteria, Firmicutes*, and *Verrucomicrobiota* after 21-day exposure to 1 mg/L of MPs (72). MPs exposure seems to enhance the expression of immune cytokines (TNF-α, IFN-γ, TLR4, and IL-6) as well as inducing microbiota dysbiosis (115). Besides aquatic animals, also terrestrial ecosystems seem to be affected by MPs. In soil animals exposed to MPs, it has been observed a remarkable reduced bacterial diversity; particularly a reduction of the abundance of *Bacteroides* and an increment of the abundance of *Firmicutes* (73). Several studies have shown that polystyrene MP can gut dysbiosis in GM and intestinal barrier dysfunction (promoting inflammation) besides metabolic disorders, including hepatic lipid metabolism, in the mice model (116, 117). In the same *in-vivo* model, polyethylene MPs were suggested to cause intestinal dysbiosis and inflammation. Particularly it was observed a significant increment in *Staphylococcus* abundance and a significant reduction in Parabacteroides abundance. Furthermore, serum levels of interleukin-1α were found significantly raised (74). Maternal MPs exposure resulted associated with GM dysbiosis and gut barrier impairment in mice, with long-term metabolic penalties in offspring (118). It was observed that polystyrene microplastics could decrease α-diversity of GM of bees and alter the expression of antioxidative, detoxification, and immune system-related genes (75). In soil containing MPs, the gut of earthworms can be damaged. Polypropylene MPs exposure can reduce diversity and alter microbial community in earthworms GM. Specifically, an increment in *Aeromonadaceae* and *Pseudomonadaceae* was observed with a decline of *Nitrososphaeraceae* and *Proteobacteria* (76). MPs can reach the intestine trait and accumulate interacting with GM and altering its composition. This toxicology assessment may represent a new target to evaluate the health hazards for humans in the future.

## Conclusion and Perspectives

The GM plays a multifaceted role in the exposure to toxic compounds. It represents the first interface between an exogenous chemical. Hence, the GM could represent a key factor in the toxicity of environmental pollutants and this may become really relevant for the identification of both novel biomarkers of exposure and molecular pathways underneath, therefore representing an indication of the true association of the exposure to the health effect observed. In this review, it has been discussed the emerging dual role played by the GM in the metabolisms of toxicants. The active modulation of the GM (e.g., with the administration of specific probiotics) to preserve the beneficial species able to neutralize the toxicity of such chemicals, may be explored in the future as a preventive therapeutic integrated approach to actively counteract exposure damages and detrimental health consequences.

In occupational medicine, a toxicological approach should be considered to elucidate GM alterations relating to environmental exposure to pollutants (119). GM composition together with the determination of well-known biomarkers could be helpful tools to assess susceptibility for disease. The risk assessment should consider that common human exposures to toxic compounds occur at low doses for a long time, while in most experimental studies, the exposure occurs for a short time at acute or sub-acute doses. It could be a new useful approach to analyze microbiome composition from a fecal sample as a screening tool, to assess the individual microbiome signature in order to address the specific intervention for preventive measures amelioration toward specific risk factors. Therefore, from a translational point-of-view, the GM may represent an important indicator for toxicological assessment, and future studies in clinics, especially in exposed cohorts of individuals, could identify the human GM as a helpful tool for the early surveillance of host health.

## Author Contributions

CF, FG, and MT contributed to the conceptualization. CC and CF contributed to the methodology and supervision. FG and MT contributed to the data curation and writing—original draft preparation. CC contributed to writing—review and editing. All authors have read and agreed to the published version of the manuscript.

## Conflict of Interest

The authors declare that the research was conducted in the absence of any commercial or financial relationships that could be construed as a potential conflict of interest.

## Publisher's Note

All claims expressed in this article are solely those of the authors and do not necessarily represent those of their affiliated organizations, or those of the publisher, the editors and the reviewers. Any product that may be evaluated in this article, or claim that may be made by its manufacturer, is not guaranteed or endorsed by the publisher.
